# Upregulation of interleukin-19 in saliva of patients with COVID-19

**DOI:** 10.1038/s41598-022-20087-w

**Published:** 2022-09-26

**Authors:** Fatemeh Saheb Sharif-Askari, Narjes Saheb Sharif-Askari, Shirin Hafezi, Swati Goel, Hawra Ali Hussain Alsayed, Abdul Wahid Ansari, Bassam Mahboub, Saleh Al-Muhsen, Mohamad-Hani Temsah, Qutayba Hamid, Rabih Halwani

**Affiliations:** 1 Sharjah Institute of Medical Research, University of Sharjah, Sharjah, United Arab Emirates; 2 Pharmacy Department, Dubai Health Authority, Dubai, United Arab Emirates; 3 Dermatology Institute, Translational Research Institute, Hamad Medical Corporation, Doha, Qatar; 4 Rashid Hospital, Dubai Health Authority, Dubai, United Arab Emirates; 5 Immunology Research Laboratory, Department of Pediatrics, College of Medicine, King Saud University, Riyadh, Saudi Arabia; 6 Department of Clinical Sciences, College of Medicine, University of Sharjah, Sharjah, United Arab Emirates; 7 Meakins-Christie Laboratories, Research Institute of the McGill University Health Center, Montreal, QC Canada; 8 Prince Abdullah Ben Khaled Celiac Disease Chair, Department of Pediatrics, Faculty of Medicine, King Saud University, Riyadh, Saudi Arabia

**Keywords:** Immunology, Cytokines, Interleukins

## Abstract

Cytokines are major players in orchestrating inflammation, disease pathogenesis and severity during COVID-19 disease. However, the role of IL-19 in COVID-19 pathogenesis remains elusive. Herein, through the analysis of transcriptomic datasets of SARS-CoV-2 infected lung cells, nasopharyngeal swabs, and lung autopsies of COVID-19 patients, we report that expression levels of IL-19 and its receptor, IL-20R2, were upregulated following SARS-CoV-2 infection. Of 202 adult COVID-19 patients, IL-19 protein level was significantly higher in blood and saliva of asymptomatic patients compared to healthy controls when adjusted for patients’ demographics (P < 0.001). Interestingly, high saliva IL-19 level was also associated with COVID-19 severity (P < 0.0001), need for mechanical ventilation (P = 0.002), and/or death (P = 0.010) within 29 days of admission, after adjusting for patients’ demographics, diabetes mellitus comorbidity, and COVID-19 serum markers of severity such as D-dimer, C-reactive protein, and ferritin. Moreover, patients who received interferon beta during their hospital stay had lower plasma IL-19 concentrations (24 pg mL^−1^) than those who received tocilizumab (39.2 pg mL^−1^) or corticosteroids (42.5 pg mL^−1^). Our findings indicate that high saliva IL-19 level was associated with COVID-19 infectivity and disease severity.

## Introduction

Interleukin (IL)-19, a member of IL-10 family of cytokines, has been studied in the context of a number of inflammatory conditions^1–4^. IL-19 is mostly produced by macrophages and B cells^5^. Airway epithelial cells (AECs) were also found to secrete IL-19 in response to stimulation with different proinflammatory cytokines including IL-4, IL-13, and IL-17^6^. IL-19 signals through its heterodimeric receptor complex, IL-20R1 and IL-20R2, and primarily activates signal transducer and activator of transcription-3 (STAT3) pathway^5,7^; which is essential for the differentiation of Th-17 and Th-2 inflammatory cells^8,9^. It is mostly produced by macrophages and B cells^5^. Airway epithelial cells (AECs) were also found to secrete IL-19 in response to stimulation with different proinflammatory cytokines including IL-4, IL-13, and IL-17^6^. The consequent activation of STAT3 was reported to contribute to pathogenesis of COVID-19 both in the early stages of SARS-CoV-2 infection, through suppression of anti-viral interferon response leading to virus spread^10,11^, and in the later stages by contributing to cytokine storms^12^.

Little is known about the role of IL-19 following bacterial or viral infections. In vivo*,* the level of IL-19 was reported to increase in relation to entry of bacteria or microbial-derived products to blood stream following the intestinal epithelial barrier breach in colitis mouse model^13^. IL-19 was shown to be induced locally in the brain tissue of mice following the intracranial challenge with bacteria^14^. IL-19 was also induced in murine colonic macrophages following treatment with bacterial liposaccharide (LPS)^13^, and in murine astrocytes following exposure to different bacterial pathogens as well as poly I:C (mimicking viral infections)^14^. However, the expression of IL-19 in the context of COVID-19 and relative to disease severity has never been investigated.

Here, we assessed the gene expression level of IL-19 in SARS-CoV-2 infected lung cells, nasopharyngeal swabs and lung autopsies of COVID-19 patients using publically available gene expression data sets. The level of IL-19 protein was also evaluated in the plasma and saliva of recruited COVID-19 patients with different levels of disease severity. Our findings indicate that IL-19 is upregulated significantly in plasma and saliva of patients with COVID-19 relative to disease severity.

## Methods

**Figure 1:**
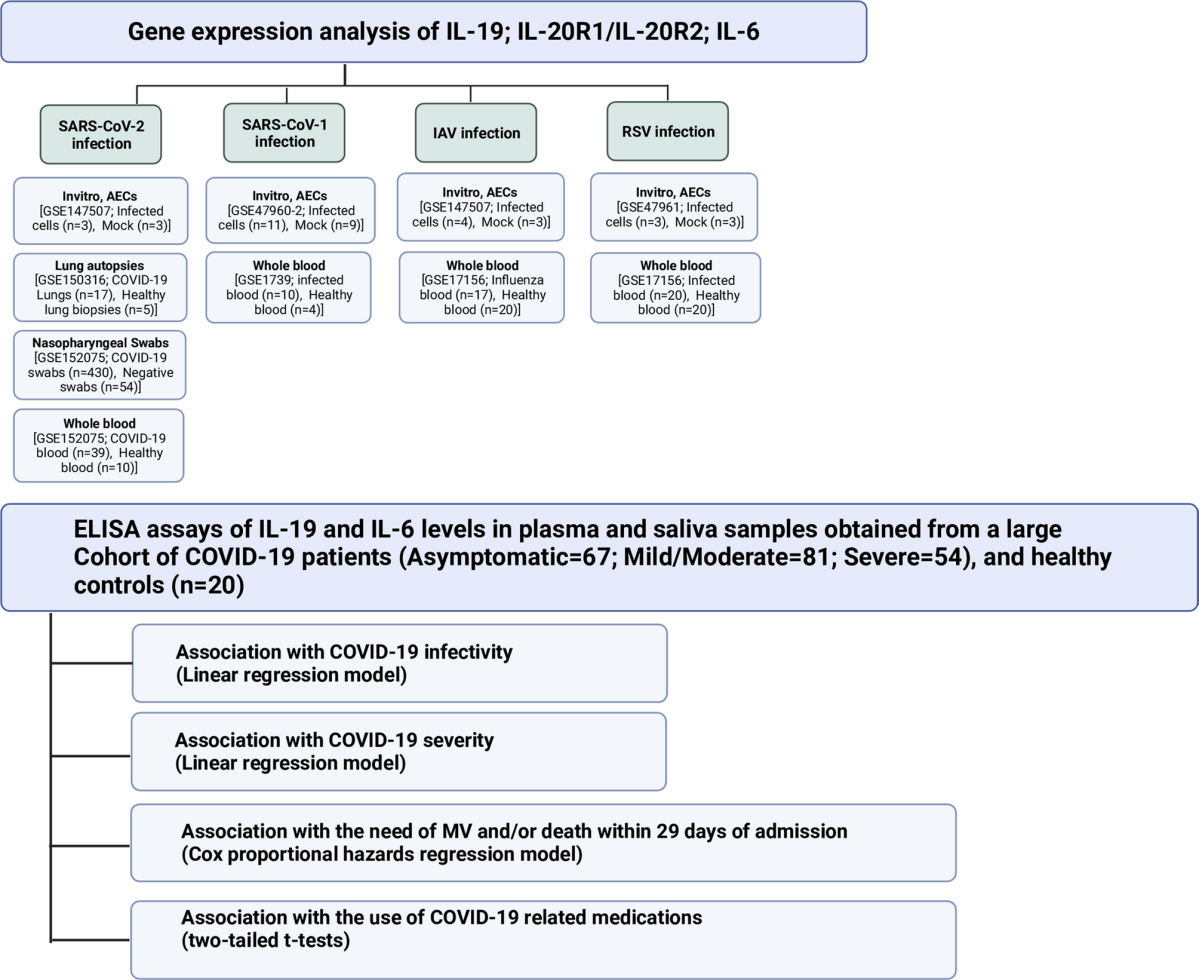
Flow chart of the study.

Gene expression level of IL-19 was evaluated in transcriptomic data sets of human airway epithelial cells (AECs) following infection with SARS-CoV-2, nasopharyngeal swabs, and lung autopsies of COVID-19 patients. Furthermore, its expression was also determined in data sets of SARS-CoV-1, influenza (IAV), and respiratory syncytial virus (RSV) infected AECs, as well as the blood of infected patients. Findings from the analysis of these datasets were confirmed by determining the protein levels of IL-19 in plasma and saliva samples of a cohort of COVID-19 patients recruited at Rashid Hospital, Dubai, UAE, using ELISA assay (Fig. 1).

### Gene expression data sets

All the gene expression data sets used for this study are available at the National Center for Biotechnology Information Gene Expression Omnibus (NCBI GEO, http://www.ncbi.nlm.nih.gov/geo) or the European Bioinformatics Institute (EMBL-EBI, https://www.ebi.ac.uk), and the details of each data set used are deposited in Supplementary Table 1.

To evaluate the effect of infection with SARS-CoV-2, or other respiratory viruses, on the expression of IL-19, the following gene expression datasets were used; COVID-19 nasopharyngeal swabs (GSE152075)^15^, SARS-CoV-2 infected lung autopsies (GSE150316)^16^, COVID-19 whole blood (EGAS00001004503)^17^, SARS-CoV-2 infected AECs (GSE147507)^18^, and other data sets of respiratory infection in AECs (SARS-CoV-1 infected AECs GSE47960, GSE47961, and GSE47962^19^; Influenza A (IAV) infected AECs, GSE147507^18^; RSV infected AECs, GSE6802^20^) or blood of infected patients (SARS-CoV-1 infected blood, GSE1739^21^; IAV and RSV infected blood, GSE17156^22^). For the COVID-19 nasopharyngeal swabs dataset (GSE152075)^15^, the investigators extracted RNA from nasopharyngeal swabs in viral transport media from 430 individuals with SARS-CoV-2 and 54 negative controls. For the COVID-19 lung autopsies dataset (GSE150316)^16^, the RNA was extracted from Formalin fixed paraffin embedded lung tissues of 52 COVID-19 fatal cases, and 5 SARS-CoV-2-uninfected individuals. For both SARS-CoV-2 and SARS-CoV-1 infections, the primary human airway epithelial cells were cultured at the air–liquid interface, as described previously, and then infected with the corresponding viruses^18,19^.

Furthermore, to assess the effect of medications on IL-19 level, we used the following gene expression data sets: BEAS-2B cells treated with or without budesonide (GSE115830), lung biopsies of patients treated with inhaled budesonide (GSE83233)^23^, peripheral blood mononuclear cells (PBMCs) of rheumatoid patients before and after treatment with tocilizumab (GSE35455) and PBMCs of multiple sclerosis patients treated with IFNβ (GSE138064)^24^.

### COVID-19 cohort

We collected plasma and saliva samples from 202 adult patients with PCR-confirmed SARS-CoV-2 infection who were referred to Rashid Hospital in Dubai between May 28 and June 30, 2020. The samples were collected on diagnosis of COVID-19 from non-hospitalized patients (asymptomatic or those with mild clinical manifestations) and early on admission from hospitalized patients. Laboratory and clinical data were collected from all patients at the time of samples collection. Patients with severe COVID-19 were followed up for 29 days after the date of hospital admission. The COVID-19 severity status was defined as COVID-19 pneumonia requiring high-flow oxygen therapy^25^. In the samples collected from 202 patients, ELISA (enzyme-linked immunosorbent assay) was used to measure level of IL-19 and other cytokine known to contribute to COVID-19 related pathogenic inflammation—IL-6—and assessed their levels with severity and patient survival^26^. Cytokine levels were also measured in the saliva of 20 healthy donors who served as control. Patient characteristics are listed in Table 1. This study was approved by the Dubai Scientific Research Ethics Committee (DSREC) and prior written informed consent was taken from all the participants of this study. All methods were performed in accordance to the relevant guidelines (Declaration of Helsinki and the Belmont Report) and regulations (DSREC rules). Precautions recommended by CDC for safe collection, handling and testing of biological fluids were followed^27^.

**Table 1:** Clinical parameters of COVID-19 patients in according to disease severity.

Variables	Healthy controls (n = 20)	COVID-19 patients			p-value
		Asymptomatic (n = 67)	Mild/moderate (n = 81)	Severe (n = 54)	
Age (years, mean, range)	32 (28–36)	33 (31–35)	48 (45–51)	57 (53–61)	< 0.0001
Gender (n, m/f)	8/12	47/20	66/15	44/9	< 0.0001
BMI (mean, range)	26 (25–27)	26 (24–27)	29 (25–33)	28 (27–30)	0.186
Salivary flow rate	0.43 ± 0.13	0.42 ± 0.18	0.39 ± 0.21	0.37 ± 0.19	0.342
**Serum inflammatory markers**					
D-dimer (0–0.5 µ mL^−1^)	–	0.27 (0.17–1.29)	0.71 (0.38–1.65)	4.73 (1.23–10.30)	< 0.0001
CRP (1.0–3.0 mg L^−1^)	–	1.25 (0.45–19)	19.2 (3–100.1)	73.60 (17.3–141.6)	0.002
Ferritin (10–204 ng mL^−1^)	–	45.2 (37–75)	535 (234–1197)	886 (465.8–1612.4)	< 0.0001
**Cytokines values**					
Plasma IL-19, pg mL^−1^	8.5 (8.2–9.2)	22.3 (18–32)	34.5 (26–56)	61.8 (44–84)	< 0.0001
Saliva IL-19, pg mL^−1^	102.1 (96–121)	533.2 (402–703)	711.9 (526–827)	862.3 (690–1054)	< 0.0001
Plasma IL-6, pg mL^−1^	50.4 (40–53)	53.3 (34–74)	106.4 (67–155)	239.2 (90–319)	< 0.0001
Saliva IL-6, pg mL^−1^	15.4 (10–20)	20.6 (9–39)	29.2 (9.8–53)	147.8 (87–222)	< 0.0001
**Comorbidity**					
DM (n, %)	1 (1)	2 (3)	39 (48)	27 (54)	< 0.0001
**COVID-19 supportive medication**					
Corticosteroids	0	0	25 (30.9)	48 (90.6)	
Tocilizumab	0	0	10 (12.3)	23 (43.4)	
IFN-α/β	0	0	22 (27.2)	24 (45.3)	

*BMI* body mass index, *CRP* C-reactive protein. Detection limits for ELISA assay of IL-19 is 7.81 pg mL^−1^, and IL-6 is 9.38 pg mL^−1^.

### Collection of saliva

For saliva collection, we followed the unstimulated whole saliva collection method^28^. Prior to saliva collection, participants were asked to sit upright and with their head slightly tilted downward to allow the saliva to collect on the floor of the mouth. The first sample was discarded to eliminate the unwanted substances and food debris contaminating the sample. The subsequent saliva sample (around 2 mL) was then dribbled into a pre-labeled polypropylene sterile tube. For each participant, salivary flow rate was calculated by dividing the saliva volume (mL) by the collection time (min)^29^. The volume of saliva was determined by weighing, assuming a density of 1 g mL^−1^ for saliva. All samples were then stored at – 20 °C until immediately before use.

### ELISA assay of IL-19 and IL-6 cytokines

IL-19 and IL-6 cytokine concentrations were determined in plasma and saliva samples using commercially available human ELISA kit (Human IL-6, DY206-05, R&D; and human IL-19 ELISA KIT, ab231922, Abcam). For the assay, saliva samples were centrifuged at 700×*g* for 15 min at 4 °C, and the supernatant was used. Assays were preformed following the manufacturer’s instructions. All samples were measured in duplicates.

### Western blot assay

Saliva samples were pelleted by centrifugation at 14,000×*g* for 15 min at 4 °C. The concentration of protein were measured using the Pierce BCA assay (Thermo-Scientific, IL, USA). Cells were lysed by 10 × RIPA lysis Buffer (Abcam, UK) supplemented with 1 × Protease Inhibitor Cocktail (cat#P8340, Sigma-Aldrich) and 1 mM phenylmethylsulfonyl fluoride (cat#78830, Sigma-Aldrich). 15 µg of total proteins were separated using 8% SDS-PAGE gels. The proteins were then transferred onto nitrocellulose membrane (Bio-Rad, Ca, USA), blocked in 5% skimmed milk for 1 h at room temperature. Blots were cut prior to hybridization with antibodies specific to p-STAT3 (cat# 9145, Cell Signaling) and STAT3 (cat# 9132, Cell Signaling). β-Actin (cat#8457, Cell Signaling) was used as loading controls. The full-length blots (uncut) of the replicates are represented in Supplementary Fig. 1. The blots were developed using the Clarity™ Western ECL Blotting Substrate (Bio-Rad, Ca, USA) in the ChemiDoc™ Touch Gel Imaging System (Bio-Rad, Ca, USA). Image Lab™ software (version 6.1, Bio-Rad, Ca, USA) was used for image acquisition and densitometric analysis of the protein bands on the blots. The software interprets the blot image in three dimensions with the length and width of the band rising as a peak out of the blot surface and measures the density of a given band as the total area under the peak^30^.

### Analysis procedures

The raw Affymetrix microarray data was normalized with Robust Multi-Array Average (RMA) and log transformed^31^. The RNA-seq data was normalized using the Bioconductor package *limma-voom* package^32^. The fold change of differentially expressed genes were conducted using *Limma* package^33,34^.

Moreover, the associations of IL-19 and IL-6 cytokine concentrations in blood and saliva of COVID-19 patients with disease severity were evaluated using linear regression models adjusted for patient’s demographic factors including age, gender, and body mass index (BMI); comorbidity such as diabetes mellitus (DM); and COVID-19 severity markers such as D-dimer, C-reactive protein (CRP), and ferritin. To avoid any strong correlation between the selected variables, all the variables were tested for the presence of collinearity using the variance inflation factors (VIF) and magnitude of standard errors.

Furthermore, the association of IL-19 and IL-6 saliva concentrations of severe COVID-19 patients with the need for mechanical ventilation and/or death within 29 days from admission was evaluated using Cox proportional hazards regression models adjusted for all the above-mentioned patient demographics and markers of COVID-19 severity. The Kaplan–Meier survival curves were then plotted to show cumulative survival over the 29-days period.

Analyses was performed using R software version 3.0.2, IBM SPSS statistics version 26, and Graphpad Prism version 7. P-values < 0.05 was considered statistically significant for all analyses.

## Results

### Enhanced IL-19 gene expression in upper and lower respiratory tract of COVID-19 patients

**Figure 2:**
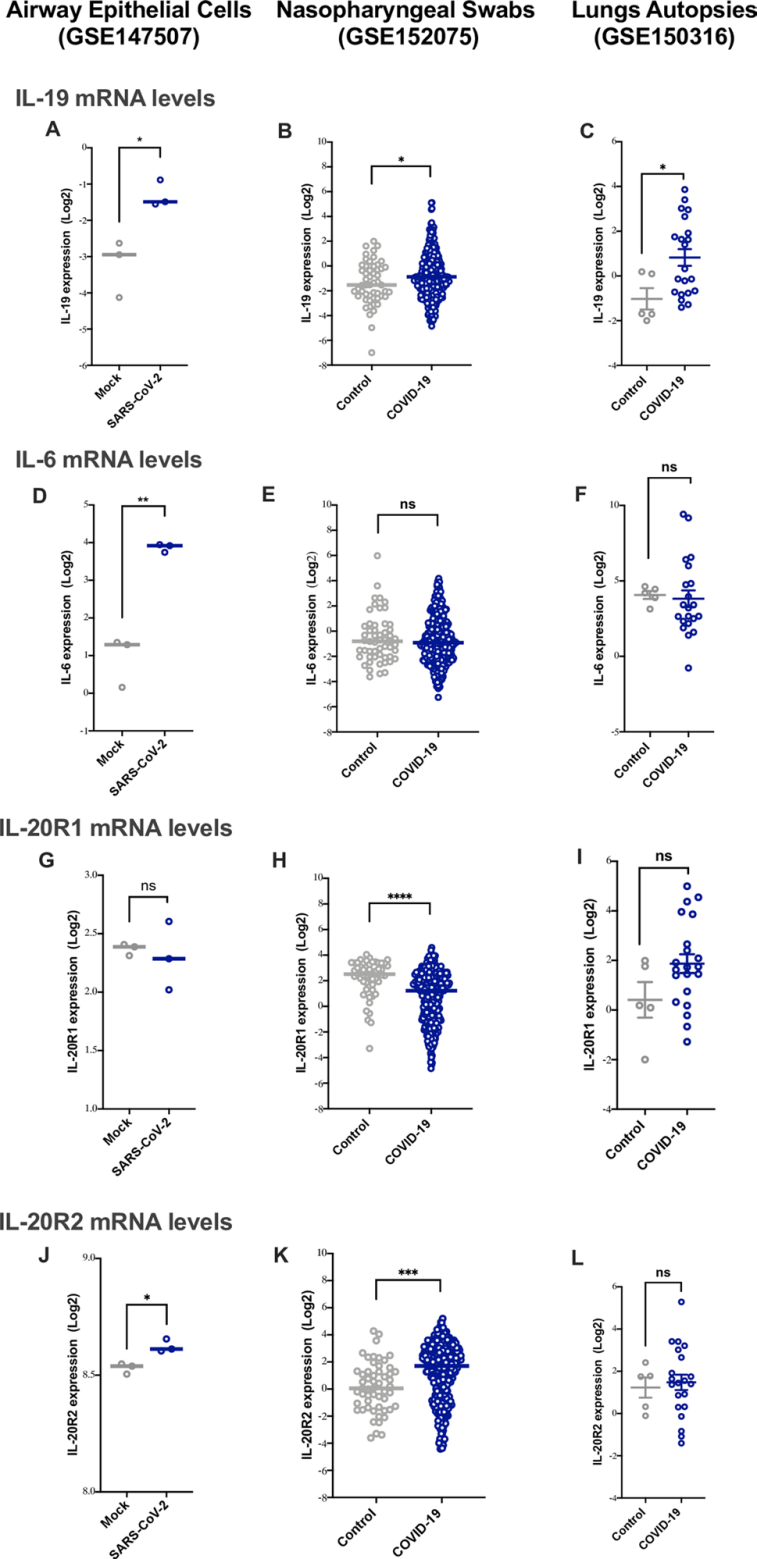
Higher IL-19 gene expression levels in lung and nasopharyngeal swabs of COVID-19 patients. (**A–C**) IL-19 mRNA levels in SARS-CoV-2 infected human airway epithelial cells [HAECs] (n = 3 infected HAECs vs. n = 3 mock-treated HAECs; GSE147507), nasopharyngeal swabs of COVID-19 patients (n = 430 COVID-19 patients vs n = 54 healthy controls; GSE152075); as well as lung autopsies of COVID-19 patients (n = 17) SARS-CoV-2 infected lung vs. n = 5 healthy lung biopsies; GSE150316). (**D–F**) IL-6 mRNA levels in SARS-CoV-2 infected HAECs, in nasopharyngeal swabs of COVID-19 patients as well as lung autopsies of COVID-19 patients. (**G–L**) IL-20R1 and IL-20R2 (IL-19 heterodimer receptors) mRNA levels in SARS-CoV-2 infected HAECs, in nasopharyngeal swabs as well as lung autopsies of COVID-19 patients. Comparison was done using unpaired t-test or Mann–Whitney *U* test, depending on the skewness of the data. ns = non-significant, *P < 0.05, **P < 0.01, ***P < 0.001, ****P < 0.0001.

To assess the level of IL-19 in the airways following SARS-CoV-2 infection, we evaluated IL-19 gene expression levels in vitro, in SARS-CoV-2 infected AECs, and in vivo, in nasopharyngeal swabs and lung autopsies of COVID-19 patients. Notably, IL-19 levels in these samples were significantly upregulated following the infection. (Fig. 2A, Log Fold-Change (FC) of 1.9 ± 0.05, in SARS-CoV-2 infected AECs; P = 0.018, Fig. 2B, logFC of 0.5 ± 0.2, in COVID-19’s nasopharyngeal swabs; P = 0.038; Fig. 2C, LogFC of 1.8 ± 0.8, in COVID-19’s lung autopsies; P = 0.031).

In contrast, the expression level of IL-6 was only upregulated in vitro, in SARS-CoV-2 infected AECs (Fig. 2D, log-fold of 2.9 ± 0.3; P = 0.001); and not in nasopharyngeal swabs or lung autopsies of COVID-19 patients (Fig. 2E,F).

Moreover, to gain better understanding of IL-19 signaling following SARS-CoV-2 infection, we examined levels of IL-19 heterodimer receptor complex, IL-20R1 and IL-20R2, in SARS-CoV-2 infected AECs, nasopharyngeal swabs and lung autopsies of COVID-19 patients (Fig. 2G–L). Interestingly, the level of IL-20R2, and not IL-20R1, was particularly higher in SARS-CoV-2 infected AECs and in nasopharyngeal swabs of COVID-19 patients (Fig. 2J, log-fold of 1.08 ± 0.2, in SARS-CoV-2 infected AECs; P = 0.0002, and Fig. 2K, log-fold of 0.1 ± 0.02, in COVID-19’s nasopharyngeal swabs; P = 0.010). Although not significant, there was an increasing trend with the level of IL-20R2 in COVID-19’s lung autopsies (Fig. 2L, P = 0.378). These results suggest that IL-20R2 receptor could be the main receptor used by IL-19 signaling following SARS-CoV-2 infection. However, further mechanistic investigation is needed to confirm this.

### Increased expression of IL-19 in saliva of SARS-CoV-2 infected patients

Next, to test whether IL-19 level correlates with SARS-CoV-2 infectivity and COVID-19 severity, we measured IL-19 protein level in the plasma and saliva of our recruited COVID-19 patients (n = 202) and heathy controls (n = 20). Out of 202 patients with PCR confirmed SARS-CoV-2 infection, 67 patients were asymptomatic, 81 patients were symptomatic with mild to moderate forms of COVID-19, and 54 had severe COVID-19 disease requiring high-flow oxygen or mechanical ventilation during hospital stay^35^. Patient characteristics are listed in Table 1.

**Figure 3:**
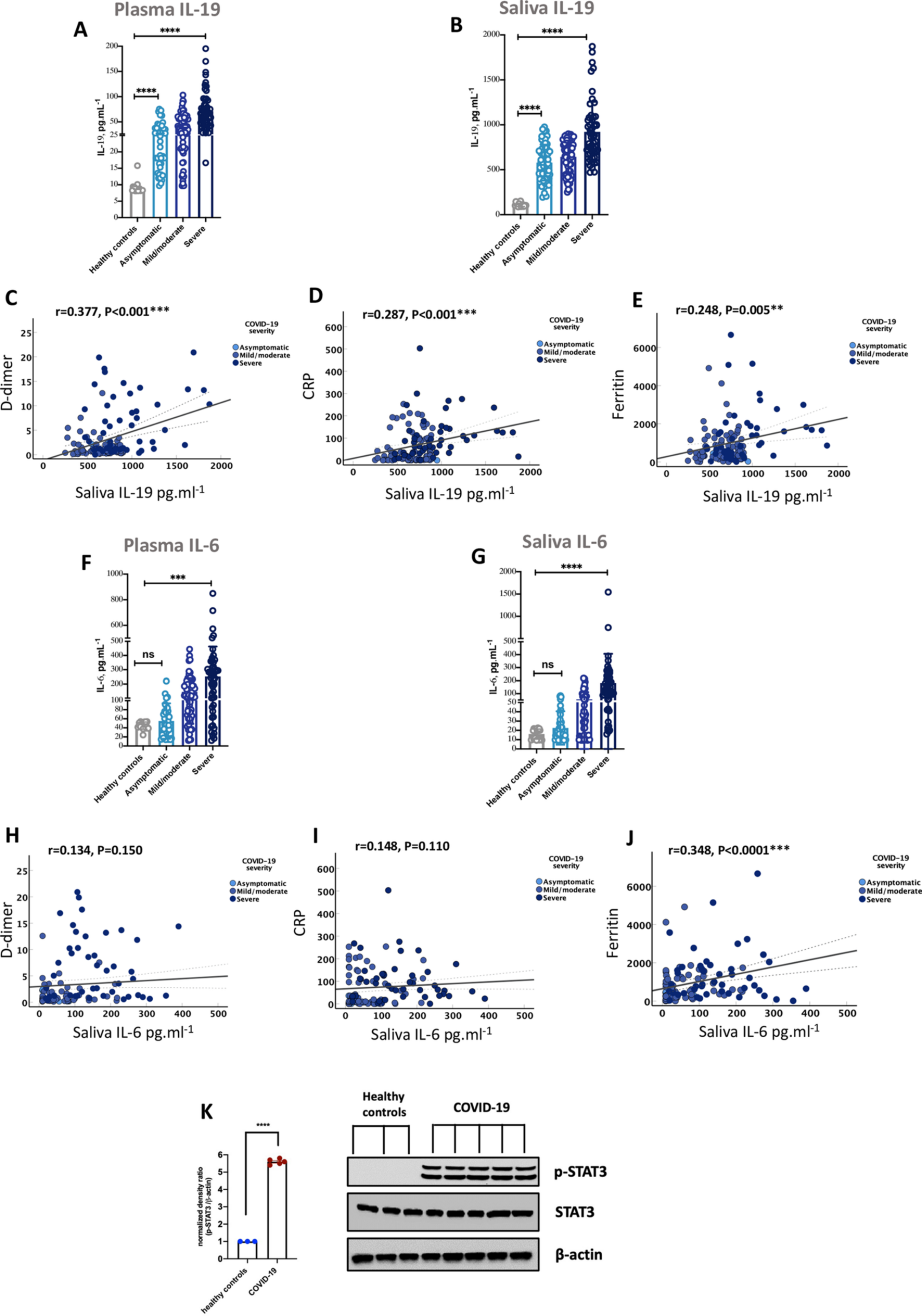
Higher IL-19 protein level in saliva of asymptomatic and severe COVID-19 patients. (**A**, **B**) IL-19 levels in plasma and saliva of COVID-19 patients with different severity (asymptomatic [n = 67], mild/moderate [n = 81], and severe [n = 54]), as well as healthy controls (n = 20). (**C–E**) Correlation of IL-19 saliva level with serum levels of D-dimer, CRP, and ferritin of these patients. (**F**, **G**) IL-6 levels in plasma and saliva of COVID-19 patients with different severity. (**H–J**) Correlation of IL-6 saliva level with serum levels of D-dimer, CRP, and ferritin of these patients. (**K**) Protein expression of p-STAT3 in saliva of COVID-19 patients (n = 5) and of healthy controls (n = 3). Blots were cut prior to hybridization with antibodies during immunoblotting. The full-length blots (uncut) of the replicates are represented in Supplementary Fig. 1. Statistical tests: Linear regression models adjusted for patient’s demographics factors (age, male sex, and body mass index), comorbidities (diabetes mellitus), and COVID-19 related severity serum markers (D-dimer, C-reactive protein, and ferritin); the Pearson correlation test; and Unpaired t-test or Mann–Whitney *U* test, depending on the skewness of the data. *ns* non-significant, *P < 0.05, **P < 0.01, ***P < 0.001, ****P < 0.0001.

IL-19 level was significantly elevated in plasma and saliva of asymptomatic COVID-19 patients compared to healthy individuals after adjustment for age, gender, and BMI (Fig. 3A,B, P < 0.001; Table 1 and Supplementary Table 2). Moreover, we noticed a significant increase in IL-19 plasma and saliva levels relative to COVID-19 severity. IL-19 plasma and saliva levels were significantly higher in severe COVID-19 patients compared to asymptomatic, or mild/moderate COVID-19 cases after adjusting for patient’s age, male sex, BMI, DM, and COVID-19 severity markers including D-dimer, CRP, and ferritin (Fig. 3A for plasma, P < 0.001; and Fig. 3B for saliva, P < 0.001; Supplementary Table 3). In addition, we correlated IL-19 level in saliva of COVID-19 patients with the serum markers of COVID-19 severity. Interestingly, IL-19 saliva level was positively correlated with serum D-dimer, CRP, and ferritin levels of these patients (Fig. 3C–E).

Next, we assessed the level of IL-6 in plasma and saliva of the COVID-19 patients. IL-6 was not associated with SARS-CoV-2 infectivity as there was no significant difference in the plasma and saliva levels of IL-6 in asymptomatic COVID-19 patients compared to healthy controls when adjusted for age, male sex, and BMI (Fig. 3F–G, P = 0.340; Table 1 and Supplementary Table 2). However, relative to COVID-19 severity, IL-6 saliva and plasma levels were associated with increased disease severity after adjusting for age, male sex, BMI, DM, and COVID-19 severity markers including D-dimer, CRP, and ferritin (Fig. 3F for plasma, P < 0.001; and Fig. 3G for saliva*,* Supplementary Table 3). Moreover, IL-6 level in saliva of COVID-19 patients was positively correlated with serum ferritin (Fig. 3J, P < 0.0001), and had a positive trend with D-dimer and CRP. (Fig. 3H,I, P  = 0.1).

Taken together, the observed increase in saliva IL-19 levels in asymptomatic COVID-19 cases compared to healthy controls, and in severe COVID-19 cases suggest the potential use of IL-19 as a non-invasive salivary diagnostic and prognostic biomarker for COVID-19 severity. Moreover, as expected, the level of phosphorylated STAT3 (p-STAT3), which is regulated by IL-19, was significantly upregulated in saliva of COVID-19 patients compared to healthy controls (Fig. 3K, Supplementary Fig. 1), which is suggestive of IL-19 signaling. However, the question of how the expression of IL‐19 is triggered during SARS-CoV-2 infection in vivo awaits further investigation.

### Higher salivary IL-19 level associated with higher risk for mechanical ventilation and/or death by 29 days after admission

**Figure 4:**
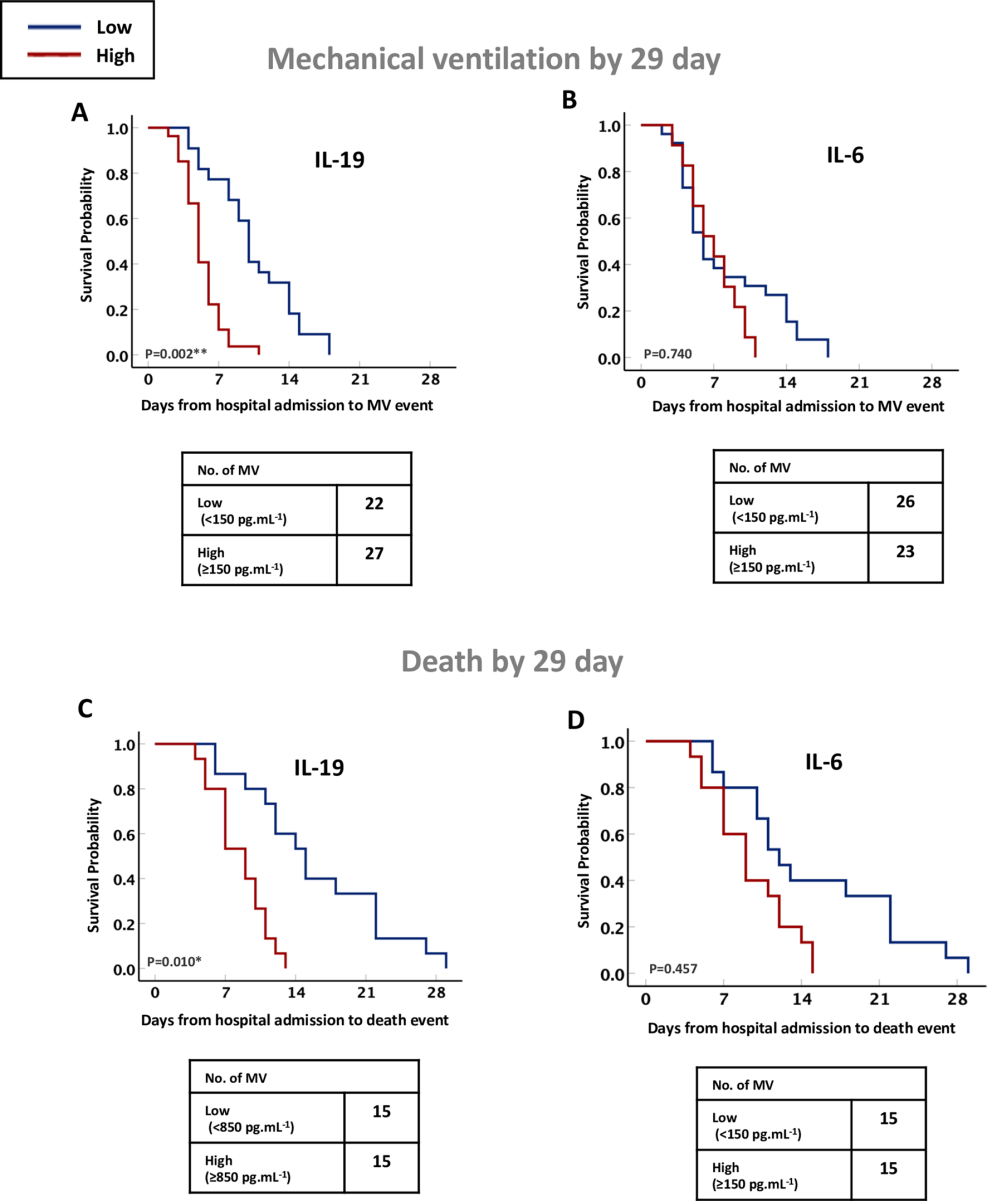
Increased IL-19 level in saliva of severe COVID-19 patients associated with higher need for mechanical ventilation and/or death by day 29. Kaplan–Meier survival curves of the need for mechanical ventilation (**A**, **B**) and/or death (**C**, **D**), based on the IL-19 or IL-6 cytokine levels in saliva of patients with severe COVID-19 (n = 54). Statistical test: Cox proportional models adjusted for patient’s demographics factors (age, male sex, and body mass index), comorbidities (diabetes mellitus), and COVID-19 related severity serum markers (D-dimer, C-reactive protein, and ferritin), with significance indicated by P value of less than 0.05.

Next, we have evaluated the association between IL-19 and IL-6 levels in saliva of severe COVID-19 cases with the survival outcomes of these patients (Fig. 4A–D). By stratifying patients into high versus low cytokine levels using the cutoffs identified in the severe COVID-19 cases, we found that high IL-19 level in saliva (≥ 850 pg mL^−1^) of severe COVID-19 cases was predictive of the need for mechanical ventilation and/or death by day 29 following admission, after adjusting for age, male sex, BMI, DM, and COVID-19 severity markers including D-dimer, CRP, and ferritin. (Fig. 4A, adjusted hazard ratio (aHR) for mechanical ventilation, 4.46; 95% CI 1.7–11.6; P = 0.002, and Fig. 4C, aHR for death, 6.39; 95% CI 1.5–26.1; P = 0.010; Supplementary Table 4). However, high IL-6 level in saliva (≥ 150 pg mL^−1^) of these patients was not predictor for worse clinical outcomes of COVID-19, after adjusting for the above-mentioned patients’ factors and COVID-19 severity markers (Fig. 4B, P = 0.740, and Fig. 4D, P = 0.457; Supplementary Table 4).

### Specific increase in IL-19 gene expression level following infection with SARS-CoV-2 but not IAV, or RSV

**Figure 5:**
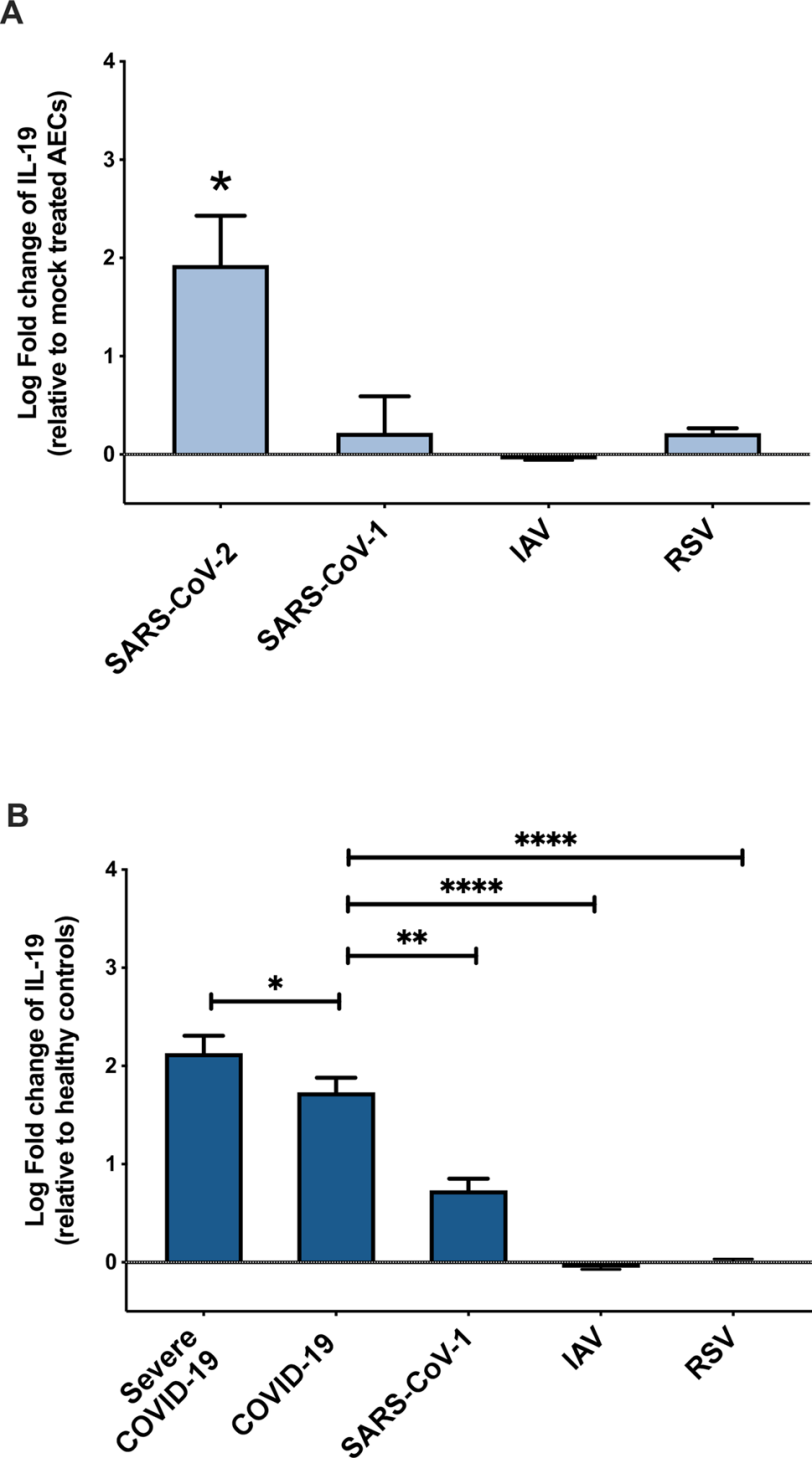
Expression of IL-19 during SARS-CoV-2 and other viral infections. (**A**) Upregulation of IL-19 in AECs infected with SARS-CoV-2 compared to other respiratory viral infections. (**B**) Upregulation of IL-19 in peripheral blood of infected patients with SARS-CoV-2 compared to other respiratory viral infections. LogFC was determined using adjusted *LIMMA*. Unpaired student t-test was used to compare between fold changes. *P < 0.05, **P < 0.01, ***P < 0.001, ****P < 0.0001.

Here, by using gene expression data sets of AECs infected with SARS-CoV-2, SARS-CoV-1, IAV, or RSV infection of AECs, we found that in vitro, IL-19 cytokine is upregulated to a higher-level during SARS-CoV-2 infection than following any other respiratory viral infections (Fig. 5A, log-fold of 1.71 ± 0.5 compared to SARS-CoV-1 and RSV; P = 0.027). Moreover, IL-19 gene expression level in vivo, was significantly higher in the blood of COVID-19 patients compared to patients with SARS-CoV-1, IAV, or RSV infection (Fig. 5B, log-fold of 0.99 ± 0.3 compared to SARS-CoV-1; P = 0.002, and log-fold of 1.7 ± 0.2 compared to IAV or RSV; P < 0.0001).

### IFNβ, but not other COVID-19 specific medications, regulate IL-19 plasma levels

**Figure 6:**
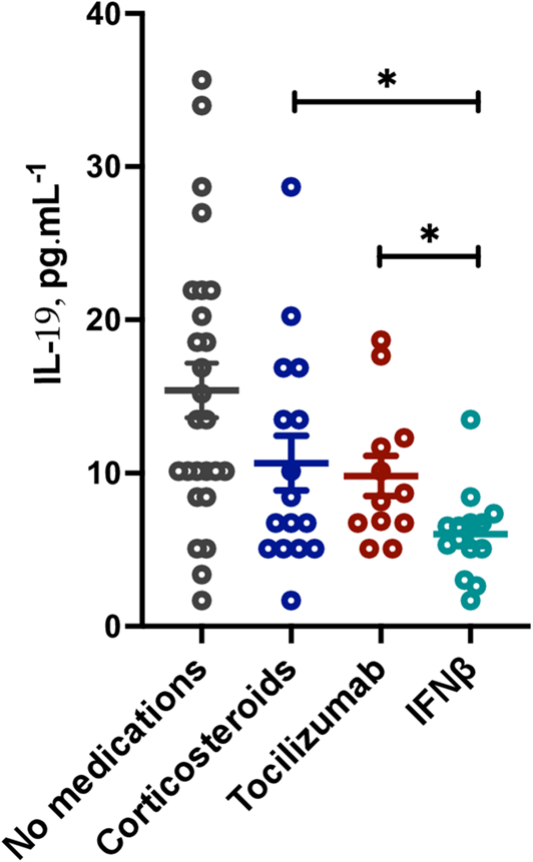
Plasma interleukin (IL)-19 levels in COVID-19 patients on different treatment regimens. Level of IL-19 in the plasma of COVID-19 patients untreated or treated with corticosteroids, tocilizumab, or IFNβ during their course of hospital treatment**.** The data show a significant reduced IL-19 plasma level of IFNβ treated patient and a trend of reduction for glucocorticoids or tocilizumab treated COVID-19 patients. Statistical tests: Unpaired t-test or Mann–Whitney *U* test, depending on the skewness of data. *NS* nonsignificant. ***P < 0.001.

We then determined whether IL-19 level are affected by common COVID-19 medications. To evaluate that, we measured the plasma IL-19 level of severe COVID-19 patients who were on corticosteroids, tocilizumab (humanized anti-IL-6 receptor antibody), or interferon-β (IFNβ) treatment. We found that patients who were on IFNβ treatment had lower IL-19 plasma level compared to those on corticosteroids or tocilizumab (Fig. 6, mean 24 ± 12.4 pg mL^−1^ in IFNβ-treated vs mean 39.2 ± 18.1 pg mL^−1^ in tocilizumab-treated, P = 0.016; and mean 42.5 ± 23.6 pg mL^−1^ in corticosteroid-treated COVID-19 patients, P = 0.031). Furthermore, using drug treatment gene expression data sets of corticosteroids, tocilizumab, and IFNβ we found that IL-19 level was not affected by corticosteroids treatment*,* in budesonide treated human AECs, or in the lung biopsies of budesonide treated individuals (Supplementary Fig. 2A,B). IL-19 gene expression level was also not affected by tocilizumab treatment in PBMCs of rheumatoid patients (Supplementary Fig. 2C). However, there was a significant reduction in IL-19 expression with IFNβ treatment in PBMCs of multiple sclerosis patients^24^ (log-fold of 1.3 ± 0.4; P = 0.004) (Supplementary Fig. 2D).

## Discussion

In this study, we show that IL-19 level is significantly increased in saliva of asymptomatic COVID-19 patients compared to healthy controls, and its level associated with COVID-19 disease severity. Furthermore, higher IL-19 level in saliva of severe COVID-19 patients was associated with higher need for mechanical ventilation and/or death within 29 days of admission. This suggests that IL-19 can be utilized as a noninvasive salivary biomarker for COVID-19 infectivity and severity.

Limited data is available regarding IL-19 regulation during viral infections. Our findings indicate that IL-19 expression pattern in SARS-CoV-2 infection is distinct from that observed in conventional respiratory viral infections such as influenza or RSV infections. IL-19 expression was higher in vitro, in SARS-CoV-2 infected AECs, and in COVID-19 patients’ blood compared to influenza or RSV infections, suggesting it may represent a marker of coronavirus infection. To further support this, IL-19 expression level correlated positively with the SARS-CoV-2 viral levels in nasopharyngeal swabs and lung autopsies of COVID-19 patients (Supplementary Fig. 3). We have previously reported that IL-19 regulates expression of ACE2, the SARS-CoV-2 entry receptor, in human primary lung cells^36^. Collectively, this may suggest IL-19 enhances susceptibility to SARS-CoV-2 infection.

IL-19 signals through IL-20R1 and IL-20R2 heterodimeric receptors, and results in activation of STAT3 inflammatory pathway^7^. The expression of IL-20R1 and IL-20R2 has been identified in different human tissues, and was highest in skin, reproductive organs, and lungs^37,38^. In fact, their expression in lung tissues would suggest a role for IL-19 during the inflammatory response to infections. In addition, our data indicated that among IL-19 receptor heterodimer, IL-20R2 was upregulated following infection of AECs with SARS-CoV-2 as well as in nasopharyngeal swabs of COVID-19 patients, suggesting that IL-20R2 could be the main receptor through which IL-19 signals during SARS-CoV-2 infection. However, further mechanistic investigation is needed to confirm this.

IL-19 level was consistently elevated both during the early phase of SARS-CoV-2 infection (in infected-AECs and COVID-19’s nasopharyngeal swabs) as well as in the acute or hyperinflammatory phase (in COVID-19’s bronchial autopsies) indicating that IL-19 actively participates in the inflammatory response to infection. Studies have suggested IL-19 and its receptors to be associated with several inflammatory conditions such as rheumatoid arthritis^1^, inflammatory bowel disease^13^, psoriasis^2^, severe asthma^3^, and severe sepsis or septic shock^4^. At the cellular level, stimulation of monocytes with IL-19 induced the production of TNFα, IL-6, and reactive oxygen species, as well as induced monocyte apoptosis^39^. Synovial fibroblasts have been reported to produce TNFα and IL-6 after stimulation with IL-19^40^. In addition, stimulation of lung epithelial cells with IL-19 resulted in the induction of IL-1β, IL-6, and IL-8 cytokines^41^. These cytokines are among the key contributors to COVID-19 cytokine storm via the activation of STAT3 and MAPK inflammatory pathways^10^. In fact, STAT3 was hyperactive in saliva of COVID-19 cases suggestive of IL-19 signaling (Fig. 3K). These findings suggest that IL-19 may play a key role in regulating the expression of inflammatory cytokines during SARS-COV-2 infection and hence eventually contribute to the cytokine storm associated with severe infections.

Previously we have shown that IL-19 was upregulated in the lungs of severe asthmatics^3^. IL-17 is known to be upregulated in the lungs of asthmatic patients relative to disease severity^42^. Here, we further observed that IL-19 expression was positively correlated with IL-17 level in nasopharyngeal swabs and lung autopsies of COVID-19 patients (Supplementary Fig. 4). This suggest that Th-17 type of lung inflammation underlies disease severity in both asthma and COVID-19. IL-17 has been shown to induce IL-19 in airway epithelial cells^6^, and hence the increased IL-19 level could be related to the elevated IL-17 level in airways of these patients^3^. In addition, APCs in response to SARS-CoV-2, induce IL-19 beside releasing IL-6, TGF-β, and IL-23 polarizing cytokines^39,43^. This in turn stimulates STAT3 signaling causing polarization and expansion of CD4^+^ T cells to Th-17 cells and production of IL-17^44^. Collectively, IL-19 as a component of the IL-23/IL-17 axis could strengthen the IL-17A-mediated immunopathological effect during severe COVID-19.

Furthermore, in this study, we showed that IFNβ significantly reduced IL-19 plasma level of COVID-19 patients, while corticosteroids and tocilizumab had limited effect on IL-19. This negative regulatory effect of IFNβ on IL-19 can be indicative of the reported STAT3 pathway attenuation following IFNβ treatment^45^. The addition of IFNβ to COVID-19 antiviral regimen might block IL-19 stimulated cytokines by downregulating IL-19 and attenuating STAT3 signaling, leading to better control of cytokine storm and the associated COVID-19 hyperinflammation condition.

While the focus of this study was only on IL-19, chosen for its known pathologic role in lung tissue^3^, additional diagnostic or predictive saliva cytokine markers will be useful. Of note, in this study, IL-6 level in saliva of COVID-19 was positively associated with ferritin and had a positive trend with other markers of COVID19 severity, D-Dimer and CRP, although not to a significant level (Fig. 3H–J, P = 0.1). This could suggest the potential use of IL-6 salivary level as a non-invasive biomarker predicting COVID-19 severity. Studies using larger cohorts, however, is needed to confirm that. All together, we believe that the results of this study will help to bring saliva cytokine measurements to standard of care for diagnosis and prognosis of COVID-19 patients.

In summary, high IL-19 level in saliva can be considered a reliable marker of COVID-19 infection and disease severity. Moreover, the value of IL-19 in saliva might help inform therapeutic interventions to determine which patients are likely to have progression of COVID-19 severity resulting in mechanical ventilation and/or death.

## Supplementary Information

Supplementary Figure 1.

Supplementary Figure 2.

Supplementary Figure 3.

Supplementary Figure 4.

Supplementary Table 1.

Supplementary Table 2.

Supplementary Table 3.

Supplementary Table 4.

## Data Availability

Al the gene expression data sets used in this study are publicly available. RMA codes. https://github.com/Bioma-85/Asthma-biomarkers/blob/main/RMA.txt. The clinical data of COVID-19 patients used are available from the corresponding author on request.

## Abbreviations

SARS-CoV-2: Severe acute respiratory syndrome-coronavirus-2
COVID-19: Coronavirus disease 2019
IL-19: Interleukin-19
IL-6: Interleukin-6
IFNβ: Interferon-beta
TGF-β: Tumor growth factor-beta
HAECs: Human airway epithelial cells
TNF-α: Tumor necrosis factor-alpha
STAT3: Signal transducer and activator of transcription-3
RSV: Respiratory syncytial virus
IAV: Influenza
PBMC: Peripheral blood mononuclear cells
