# Supplementary material for: Upregulation of interleukin-19 in saliva of patients with COVID-19
Source: Sci Rep. 2022 Sep 26;12:16019. doi: 10.1038/s41598-022-20087-w (PMC9511465; doi:10.1038/s41598-022-20087-w)
Supplement: Supplementary file 1 — Supplementary Figure 1. [file 41598_2022_20087_MOESM1_ESM.pdf]

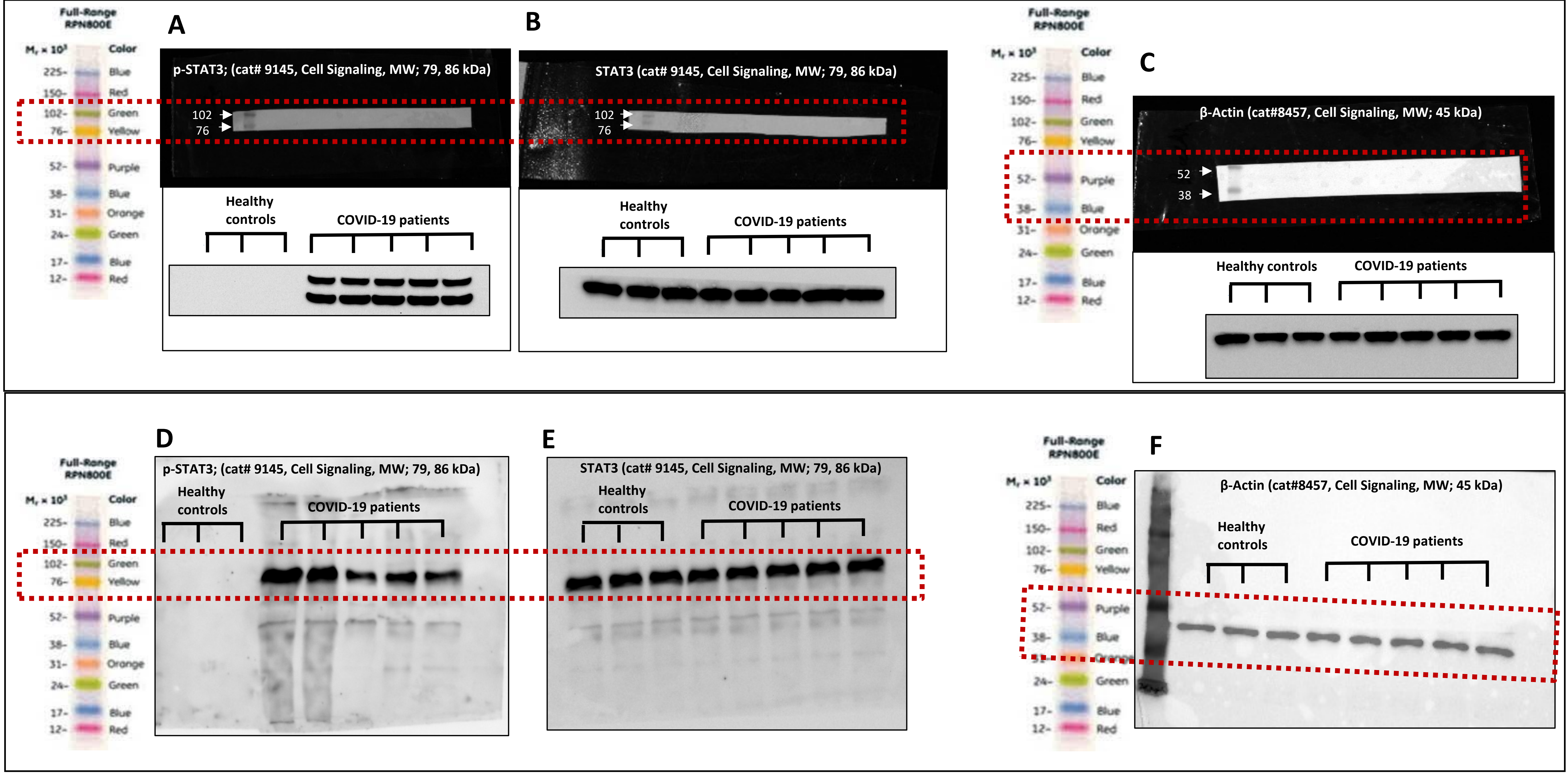

Supplementary Figure 1. Protein levels of p-STAT3, STAT3, and B-actin in saliva samples of COVID-19 patients and healthy controls. A-C; are Figure 3, and D-F are replicates. Blots were visualized on a BioRad ChemiDoc™ Touch Imager; p-STAT3 exposure time of 1 to 2 minutes (signal accumulation), STAT3 and B-actin exposure time of 10 to 30 seconds (Signal accumulation).
