# Supplementary material for: Upregulation of interleukin-19 in saliva of patients with COVID-19
Source: Sci Rep. 2022 Sep 26;12:16019. doi: 10.1038/s41598-022-20087-w (PMC9511465; doi:10.1038/s41598-022-20087-w)
Supplement: Supplementary file 2 — Supplementary Figure 2. [file 41598_2022_20087_MOESM2_ESM.pdf]

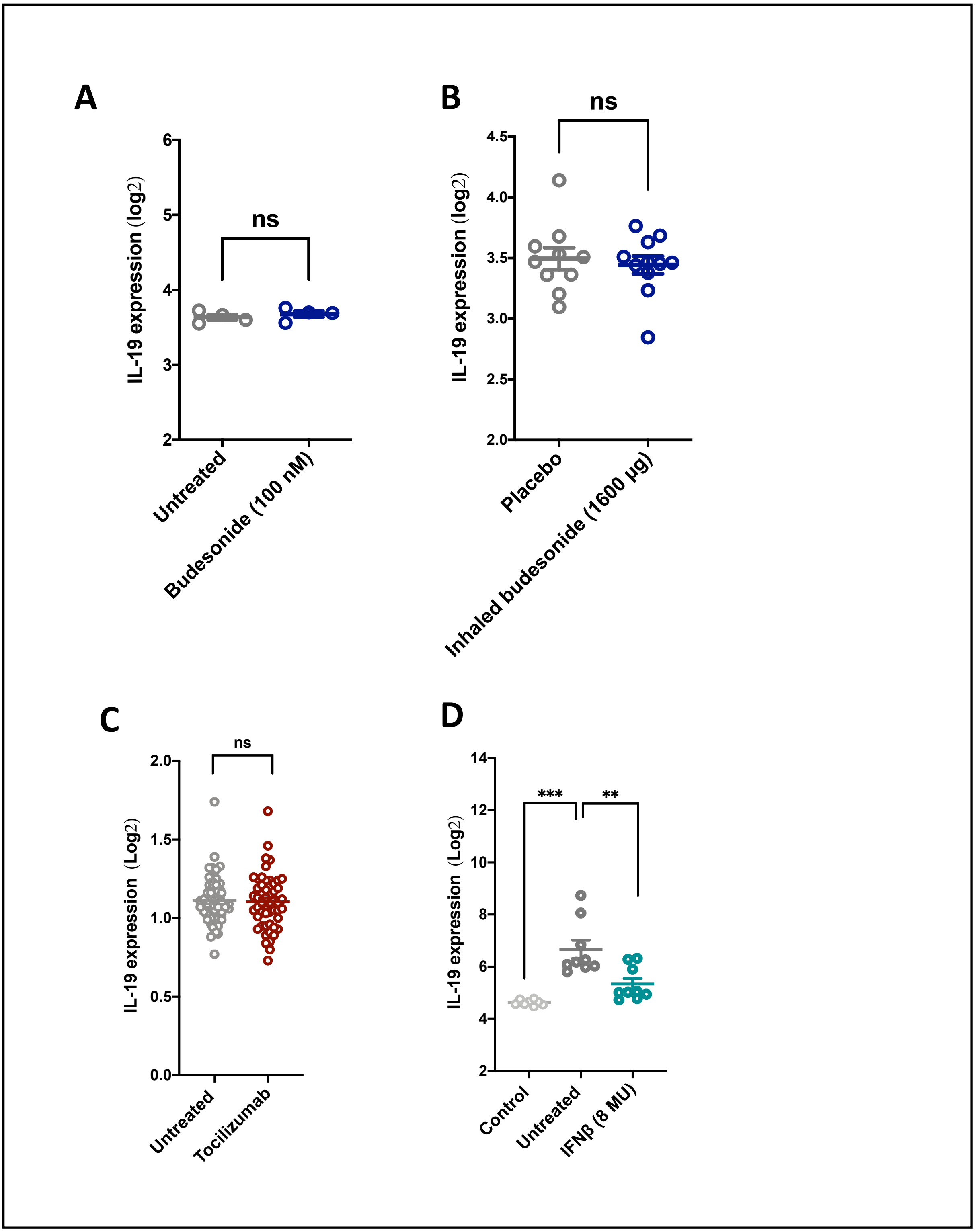

Supplementary Figure 2. Plasma interleukin (IL)-19 levels in COVID-19 patients on different treatment regimens.

A) Gene expression level of IL-19 in the human bronchial airway epithelial BEAS-2B cells untreated or treated with budesonide (100 nμ) for 18 hours. **B)** Gene expression level of IL-19 in bronchial biopsies from healthy, non-smoker, non-allergic male volunteers treated with placebo or inhaled budesonide (1600 μg). **C)** Gene expression level of IL-19 in the peripheral blood mononuclear cells (PBMCs) of rheumatoid arthritis (RA) patients untreated or treated with tocilizumab. **D)** Gene expression level of IL-19 in the PBMCs of multiple sclerosis (MS) patients untreated or treated with IFNβ**.** Statistical test: unpaired t-test or Mann-Whitney U test, depending on the skewness of the data. ns=non-significant, ** P<0.01, *** P<0.001.
