# Supplementary material for: Upregulation of interleukin-19 in saliva of patients with COVID-19
Source: Sci Rep. 2022 Sep 26;12:16019. doi: 10.1038/s41598-022-20087-w (PMC9511465; doi:10.1038/s41598-022-20087-w)
Supplement: Supplementary file 3 — Supplementary Figure 3. [file 41598_2022_20087_MOESM3_ESM.pdf]

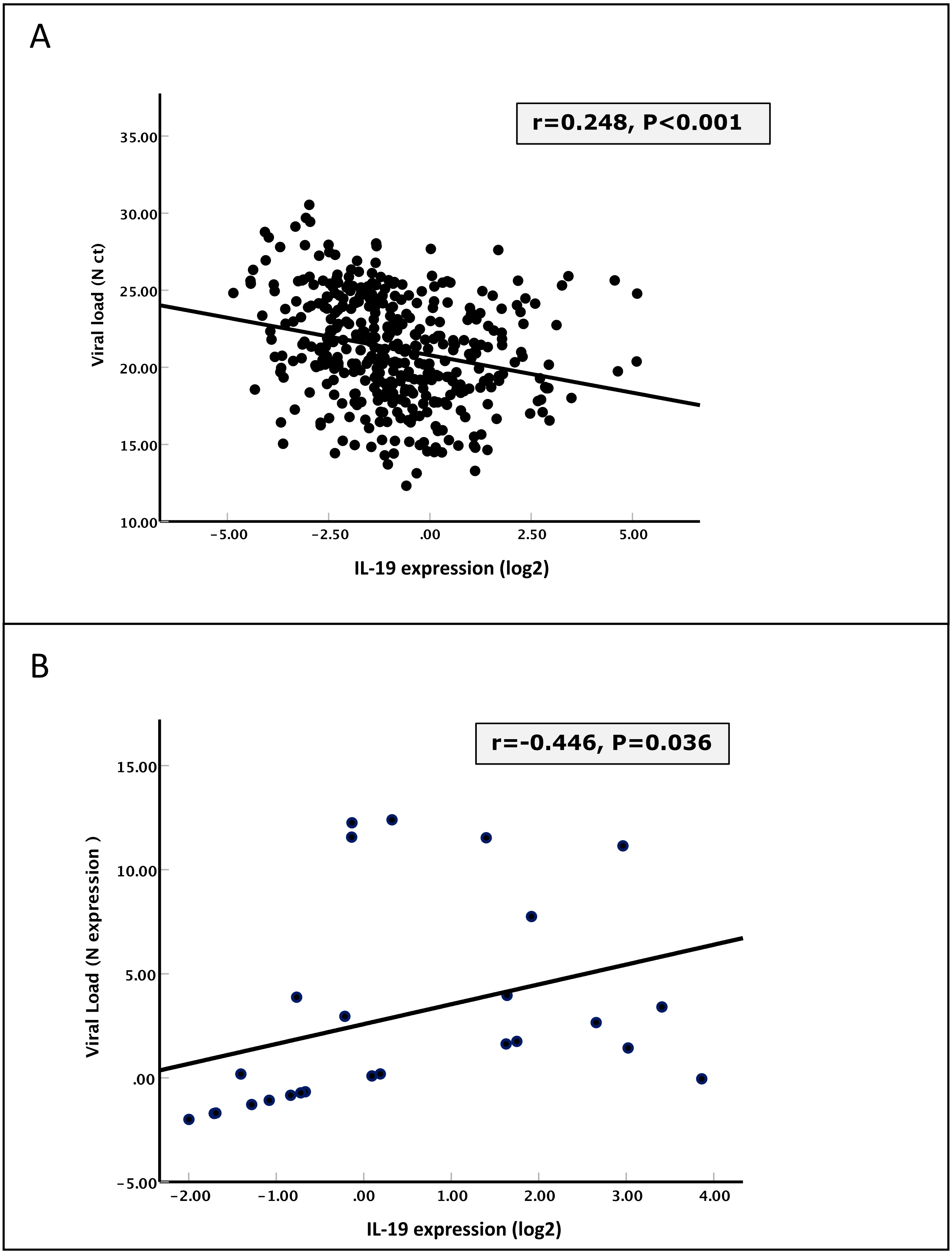

Supplementary Figure 3. Correlation between IL-19 gene expression level and SARS-CoV-2 viral loads in nasopharyngeal swabs (A) and lung autopsies of COVID-19 patients (B). Of note, the viral loads in COVID-19's nasopharyngeal were determined by RT-PCR that is, the greater the viral loads, the lower the Ct value. Statistical tests: Pearson's (A), and Spearman's rank (B) correlation coefficient tests with P-value <0.05 considered significant.
