# Supplementary material for: Upregulation of interleukin-19 in saliva of patients with COVID-19
Source: Sci Rep. 2022 Sep 26;12:16019. doi: 10.1038/s41598-022-20087-w (PMC9511465; doi:10.1038/s41598-022-20087-w)
Supplement: Supplementary file 4 — Supplementary Figure 4. [file 41598_2022_20087_MOESM4_ESM.pdf]

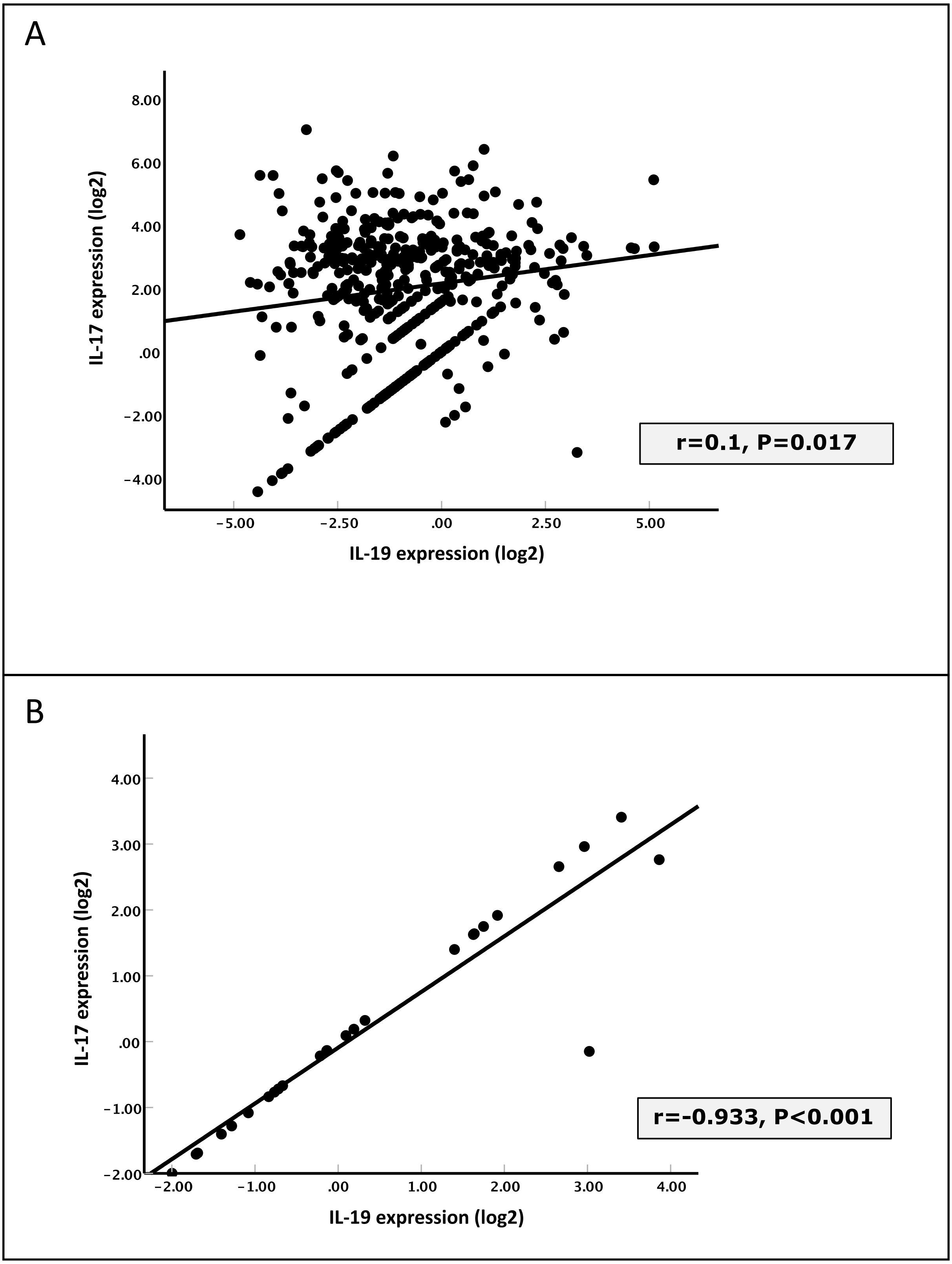

Supplementary Figure 4. Correlation between IL-17 and IL-19 gene expression levels in nasopharyngeal swabs (A) and lung autopsies of COVID-19 patients (B). Statistical tests: Pearson’s (A), and Spearman's rank (B) correlation coefficient tests with P-value <0.05 considered significant.
