# Supplementary material for: Upregulation of interleukin-19 in saliva of patients with COVID-19
Source: Sci Rep. 2022 Sep 26;12:16019. doi: 10.1038/s41598-022-20087-w (PMC9511465; doi:10.1038/s41598-022-20087-w)
Supplement: Supplementary file 5 — Supplementary Table 1. [file 41598_2022_20087_MOESM5_ESM.pdf]

Supplementary Table 1. Gene expression datasets used in this study.

Groups	Study accession No.	Platform	Cell type	Condition 1	Condition 2
**SARS-CoV-2**					
	GSE147507	GPL18573	Primary human bronchial epithelial cells	Mock (n=3)	SARS-CoV2 (n=3)
	GSE150316	GPL18573	Lung autopsies	Healthy Lung Biopsy (n=5)	COVID19 Lung (n=17)
	GSE152075	GPL18573	Nasopharyngeal Swabs	Negative controls (n=54)	SARS-CoV-2 infected (n=430)
	EGAS00001004503	GPL24676	Whole blood	Healthy controls blood (n=10)	COVID-19 blood (n=39)
**SARS-CoV1**					
	GSE47960	GPL6480	Primary human airway epithelial cells	Mock (n=3)	SARS-CoV1 (n=4)
	GSE47961	GPL6480	Primary human airway epithelial cells	Mock (n=3)	SARS-CoV1 (n=4)
	GSE47962	GPL6480	Primary human airway epithelial cells	Mock (n=3)	SARS-CoV1 (n=3)
	GSE1739	GPL201	Whole blood	Healthy controls blood (n=4)	SARS-CoV-1 blood (n=10)
**IAV**					
	GSE147507	GPL18573	Primary human airway epithelial cells	Mock (n=3)	IAV (n=4)
	GSE17156	GPL571	Whole blood	Controls (n=20)	Influenza (n=17)
**RSV**					
	GSE47961	GPL6480	Primary human airway epithelial cells	Mock (n=3)	RSV (n=3)
	GSE17156	GPL571	Whole blood	Controls (n=20)	RSV (n=20)
**Budesonide treatment**					
	GSE115830	GPL15207	BEAS-2B	Normal (n=4)	Budesonide 100 μM, time point of 18 hrs (n=4)
	GSE83233	GPL15207	Bronchial biopsy	Healthy controls (n=11)	Budesonide inhalation treated (n=11)
**Tocilizumab**					
	GSE35455	GPL1291	PBMCs of rheumatoid arthritis patients	Pre-treatment with tocilizumab (n=54)	Post-treatment with tocilizumab (n=54)
**IFNβ**					
	GSE138064	GPL17586	PBMCs of multiple sclerosis patients	Untreated patients (n=9)<br><br>Healthy controls (n=8)	IFNβ (8 MU) treated patients (n=9)
