# Supplementary material for: Upregulation of interleukin-19 in saliva of patients with COVID-19
Source: Sci Rep. 2022 Sep 26;12:16019. doi: 10.1038/s41598-022-20087-w (PMC9511465; doi:10.1038/s41598-022-20087-w)
Supplement: Supplementary file 6 — Supplementary Table 2. [file 41598_2022_20087_MOESM6_ESM.pdf]

Supplementary Table 2. Association of saliva and plasma IL-19 or IL-6 levels with asymptomatic COVID-19

**Variables**	**Adjusted (β coefficient)**	***P*-value**
Saliva IL-19 $pg.mL^{-1}$	0.783	<0.001
Plasma IL-19 $pg.mL^{-1}$	0.586	<0.001
Saliva IL-6 $pg.mL^{-1}$	0.120	0.345
Plasma IL-6 $pg.mL^{-1}$	0.122	0.379

Adjusted with patient demographic (age, gender, and BMI).
