# Supplementary material for: Upregulation of interleukin-19 in saliva of patients with COVID-19
Source: Sci Rep. 2022 Sep 26;12:16019. doi: 10.1038/s41598-022-20087-w (PMC9511465; doi:10.1038/s41598-022-20087-w)
Supplement: Supplementary file 7 — Supplementary Table 3. [file 41598_2022_20087_MOESM7_ESM.pdf]

Supplementary Table 3. Association of saliva and plasma IL-19 or IL-6 levels with severe COVID-19

Variables	Adjusted (β coefficient)	*P*-value
Saliva IL-19 $pg.mL^{-1}$	0.453	<0.001
Plasma IL-19 $pg.mL^{-1}$	0.392	<0.001
Saliva IL-6 $pg.mL^{-1}$	0.469	<0.001
Plasma IL-6 $pg.mL^{-1}$	0.360	0.002

Adjusted with patient demographic (age, gender, and BMI), comorbidities (diabetes mellitus), and serum D-dimer, C-reactive protein, and ferritin levels.
