# Supplementary material for: Upregulation of interleukin-19 in saliva of patients with COVID-19
Source: Sci Rep. 2022 Sep 26;12:16019. doi: 10.1038/s41598-022-20087-w (PMC9511465; doi:10.1038/s41598-022-20087-w)
Supplement: Supplementary file 8 — Supplementary Table 4. [file 41598_2022_20087_MOESM8_ESM.pdf]

Supplementary Table 4. Association of IL-19 or IL-6 levels in saliva of severe COVID-19 patients with the survival outcomes at 29 day of hospital admission.

Variables	Undjusted Hazard ratio	P-value	Adjusted Hazard ratio	P-value
**Need for mechanical ventilation**				
Saliva IL-19 pg.mL^{-1}	4.79 (2.3-9.9)	<0.001	4.46 (1.7-11.6)	0.002
Saliva IL-6 pg.mL^{-1}	1.48 (0.7-2.7)	0.213	0.86 (0.3-2.0)	0.740
**All-cause mortality**				
Saliva IL-19 pg.mL^{-1}	5.36 (1.9-14.5)	<0.001	6.39 (1.5-26.1)	0.010
Saliva IL-6 pg.mL^{-1}	2.53 (1.1-5.8)	0.029	1.60 (04-5.6)	0.457

Adjusted with patient demographic (age, gender, and BMI), comorbidities (diabetes mellitus), and serum D-dimer, C-reactive protein, and ferritin levels.
